# Buckling of Arteries With Noncircular Cross Sections: Theory and Finite Element Simulations

**DOI:** 10.3389/fphys.2021.712636

**Published:** 2021-08-13

**Authors:** Yasamin Seddighi, Hai-Chao Han

**Affiliations:** Department of Mechanical Engineering, The University of Texas at San Antonio, San Antonio, TX, United States

**Keywords:** mechanical instability, artery buckling, tortuosity, buckling equation, critical buckling pressure, finite element analysis

## Abstract

The stability of blood vessels is essential for maintaining the normal arterial function, and loss of stability may result in blood vessel tortuosity. The previous theoretical models of artery buckling were developed for circular vessel models, but arteries often demonstrate geometric variations such as elliptic and eccentric cross-sections. The objective of this study was to establish the theoretical foundation for noncircular blood vessel bent (i.e., lateral) buckling and simulate the buckling behavior of arteries with elliptic and eccentric cross-sections using finite element analysis. A generalized buckling equation for noncircular vessels was derived and finite element analysis was conducted to simulate the artery buckling behavior under lumen pressure and axial tension. The arterial wall was modeled as a thick-walled cylinder with hyper-elastic anisotropic and homogeneous material. The results demonstrated that oval or eccentric cross-section increases the critical buckling pressure of arteries and having both ovalness and eccentricity would further enhance the effect. We conclude that variations of the cross-sectional shape affect the critical pressure of arteries. These results improve the understanding of the mechanical stability of arteries.

## Introduction

Mechanical stability of arteries is essential for normal arterial functioning. However, tortuosity or kinking often occurs in blood vessels like coronary, carotid, or iliac arteries and veins due to high blood pressure, aging, atherosclerosis, diabetes, and other pathological changes in the arteries (Han, 2012). Previous research demonstrated that long cylindrical vessels buckle into tortuous shape when the lumen pressure exceeds its critical value (Han, 2007, 2008, 2009a,b; Goriely et al., 2008; Rachev, 2009; Lee and Han, 2010; Martinez et al., 2010; Lee et al., 2012; Dehghani et al., 2019). Artery buckling generates additional local wall stress and may impede blood flow which might result in ischemic attack to the distal organ and other vascular deficiencies (Han, 2012).

Many arteries are not ideal circular cylinders, but instead are noncircular with oval or eccentric or irregular cross-sections (Han and Fung, 1991, 1996; Aoki and Ku, 1993; MacLean and Roach, 1998; Zeina et al., 2007; Kamenskiy et al., 2012). While there is a fair understanding of how these variations affect the wall stress under lumen pressure, little is known about how they will affect the stability of arteries. Previous computational analyses showed some effects of irregular geometry such as aneurysm, stenosis, oval, or eccentric cross-section (Datir et al., 2011; Lee et al., 2014; Sanyal and Han, 2015). However, it is not clear whether the buckling equations for circular cylindrical vessels can be applied to vessels with noncircular cross-sections. Further work is needed to better understand the buckling behavior of arteries with noncircular cross-sections.

Accordingly, the objective of this study was to establish the theoretical foundation for noncircular artery buckling and to investigate the effect of concentric and eccentric oval-shaped cross-sections on the buckling behavior of arteries using finite element analysis.

## Materials and Methods

### The Theoretical Foundation for Noncircular Vessel Buckling

#### Deformation Under Lumen Pressure

**Figure 1:**
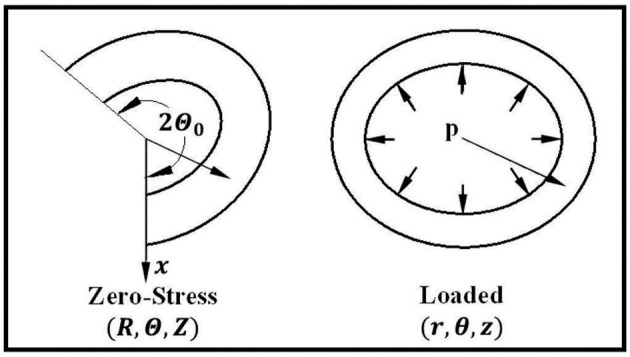
Schematics showing an artery in the zero-stress, and loaded states as well as the definitions of the coordinates. The opening angle of the artery is designated as (2π − 2Θ_0_).

Arteries were modeled as cylindrical tubes of homogenous orthotropic material with a Fung-type strain energy function (Fung, 1993). To take into account the residual stress in the arteries (Chuong and Fung, 1986; Han and Fung, 1996), we considered an artery with an opening angle of (2π − 2Θ_0_) (Figure 1). Using cylindrical polar coordinates, a material point is denoted as (*R*, Θ, *Z*) in the stress-free state (open sector) and as (*r*, θ, *z*) in the deformed state (Figure 1). Since the vessel cross-sections are noncircular, the lumen and outer radii change as functions of polar angle Θ. The initial lumen radius, outer radius, and length of the artery are designated as *R*_*i*_(Θ), *R*_*e*_(Θ), and *L*, respectively, at the zero-stress state (an open sector). Similarly, deformed inner radius, outer radius, and length at the pressure-loaded state are designated as *r*_*i*_(θ), *r*_*e*_(θ), and *l*, respectively, when it is under internal pressure *p* and axial tension *N*. The corresponding axial elongation is designated by the axial stretch ratio $\lambda_{z}^{0}$. Furthermore, the deformed polar coordinates (*r*, θ) depend on both *R* and Θ in the initial configuration.

(1) $$\begin{array}{r} r=r\left(R,\;\Theta ,p\right)\\ \theta =\theta \left(R,\;\Theta ,p\right)\\ z=\lambda_{z}^{0}Z \end{array}$$

The deformation gradient matrix ***F*** is:

(2) $$\begin{array}{l} \text{F}=\left[\begin{array}{ccc} \frac{\partial r}{\partial R} & \frac{1}{R}\frac{\partial r}{\partial \Theta } & 0\\ r\frac{\partial \theta }{\partial R} & \frac{r}{R}\frac{\partial \theta }{\partial \Theta } & 0\\ 0 & 0 & \lambda_{z}^{0} \end{array}\right] \end{array}$$

Accordingly, the Green strain tensor is:

(3) $$\begin{array}{l} \text{E}=\frac{1}{2}\left({\text{F}}^{\text{T}}\text{F}-\text{I}\right)\\ \;=\frac{1}{2}\left[\begin{array}{ccc} {\left(\frac{\partial r}{\partial R}\right)}^{2}+{\left(r\frac{\partial \theta }{\partial R}\right)}^{2}-1 & \frac{\partial r}{\partial R}\frac{1}{R}\frac{\partial r}{\partial \Theta }+r\frac{\partial \theta }{\partial R}\frac{r}{R}\frac{\partial \theta }{\partial \Theta } & 0\\ \frac{\partial r}{\partial R}\frac{1}{R}\frac{\partial r}{\partial \Theta }+r\frac{\partial \theta }{\partial R}\frac{r}{R}\frac{\partial \theta }{\partial \Theta } & {\left(\frac{1}{R}\frac{\partial r}{\partial \Theta }\right)}^{2}+{\left(\frac{r}{R}\frac{\partial \theta }{\partial \Theta }\right)}^{2}-1 & 0\\ 0 & 0 & 000{\left(\lambda_{z}^{0}\right)}^{2}-1 \end{array}\right] \end{array}$$

#### Deformation in Buckled Noncircular Arteries

**Figure 2:**
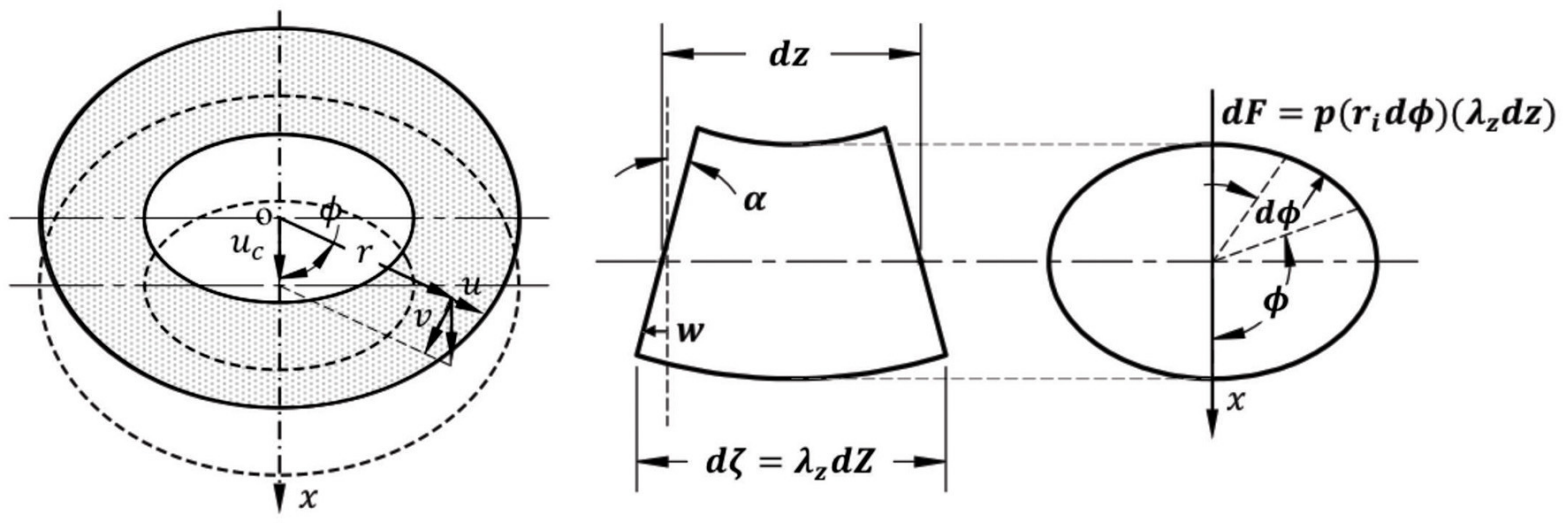
Schematic illustration of the relationship between wall displacement and central axis deflection **(left)**, a deformed segment of a buckled artery in lateral view **(middle)**, and cross-sectional view **(right)**. α represents the rotation angle of a cross-section.

When a blood vessel buckles, the central axis of the vessel deflects laterally (Figure 2). Designate the direction of lateral deflection as the x-axis and the deflection of the central axis as *u*_*c*_(*z*), where *z* is the axial coordinate of the central axis. Accordingly, the radial, circumferential, and axial displacement due to deflection *u*_*c*_(*z*) are (Han, 2008):

(4a) $$\begin{array}{l} u=u_{c}\;.\;\cos\phi \\ v={-u}_{c}\;.\;\sin\phi \\ w=-\frac{\partial u_{c}}{\partial z}\left(r.\cos\phi \right) \end{array}$$

A point (*r*, ϕ, *z*) on the arterial wall deforms into a new position (ρ, θ, ζ). The deformed radial, circumferential, and axial coordinates are (Han, 2009b; Lee et al., 2012):

(4b) $$\begin{array}{l} \rho =r\left(R,\;\Theta ,p\right)+u_{c}\;.\;\cos\phi \\ \theta =\phi \left(R,\;\Theta ,p\right)-\frac{u_{c}}{r}\sin\phi \\ \zeta =z-\frac{\partial u_{c}}{\partial z}r\cos\phi =\lambda_{z}^{0}Z-\frac{\partial u_{c}}{\partial z}r\cos\phi \end{array}$$

Where (*R*, Θ, *Z*) are the initial polar coordinates of the point in the unloaded configuration and $\lambda_{z}^{0}$ is the axial stretch ratio. Accordingly, the deformation gradient ***F*** after neglecting the higher-order terms of *u*_*c*_ is:

(5) $$\begin{array}{l} \text{F}=\left[\begin{array}{ccc} \frac{\partial r}{\partial R}-\left(u_{c}\sin\phi \right)\frac{\partial \phi }{\partial R} & \frac{1}{R}\frac{\partial r}{\partial \Theta }-\left(u_{c}\sin\phi \right)\frac{1}{R}\frac{\partial \phi }{\partial \Theta } & \left(\frac{\partial u_{c}}{\partial z}\cos\phi \right)\frac{\partial z}{\partial Z}\\ r\frac{\partial \phi }{\partial R}+\frac{u_{c}}{r}\sin\phi \frac{\partial r}{\partial R} & \frac{r}{R}\frac{\partial \phi }{\partial \Theta }+\frac{u_{c}}{rR}\sin\phi \frac{\partial r}{\partial \Theta } & -\left(\frac{\partial u_{c}}{\partial z}\sin\phi \right)\frac{\partial z}{\partial Z}\\ \frac{\partial u_{c}}{\partial z}\left(r\sin\phi \frac{\partial \phi }{\partial R}-\cos\phi \frac{\partial r}{\partial R}\right) & \frac{\partial u_{c}}{\partial z}\frac{1}{R}\left(r\sin\phi \frac{\partial \phi }{\partial \Theta }-\cos\phi \frac{\partial r}{\partial \Theta }\right) & \left(1-\frac{\partial^{2}u_{c}}{\partial z^{2}}r\cos\phi \right)\frac{\partial z}{\partial Z} \end{array}\right] \end{array}$$

Therefore, the Green strain tensor after neglecting the higher-order terms of *u*_*c*_ is:

(6) $$\begin{array}{l} \text{E}=\frac{1}{2}\left[\begin{array}{ccc} {\left(\frac{\partial r}{\partial R}\right)}^{2}+{\left(r\frac{\partial \phi }{\partial R}\right)}^{2}-1 & \frac{1}{R}\frac{\partial r}{\partial R}\frac{\partial r}{\partial \Theta }+\frac{r}{R^{2}}\frac{\partial \phi }{\partial R}\frac{\partial \phi }{\partial \Theta } & 0\\ \frac{1}{R}\frac{\partial r}{\partial R}\frac{\partial r}{\partial \Theta }+\frac{r}{R^{2}}\frac{\partial \phi }{\partial R}\frac{\partial \phi }{\partial \Theta } & {\left(\frac{r}{R}\frac{\partial \phi }{\partial \Theta }\right)}^{2}+{\left(\frac{1}{R}\frac{\partial r}{\partial \Theta }\right)}^{2}-1 & 0\\ 0 & 0 & {\left(\frac{\partial z}{\partial Z}\right)}^{2}\left(1-2\frac{\partial^{2}u_{c}}{\partial z^{2}}r\cos\phi \right)-1 \end{array}\right] \end{array}$$

Comparing this equation with Equation (3) yields that the only nonzero incremental strain component is:

(7) $$\begin{array}{l} △E_{z}=-{\left(\frac{\partial z}{\partial Z}\right)}^{2}\frac{\partial^{2}u_{c}}{\partial z^{2}}\;r\cos\phi =-{\left(\lambda_{z}^{0}\right)}^{2}\frac{\partial^{2}u_{c}}{\partial z^{2}}\;r\cos\phi \end{array}$$

which is the axial component of the Green strain tensor. This expression for noncircular vessels is the same as for circular vessels (Lee et al., 2012).

#### The Lateral Load in Buckled Noncircular Arteries

When the vessel buckles, the deflection generates uneven lumen surface areas at the concave and convex sides of the vessel (Figure 2). Consequently, the lumen pressure *p* generated a laterally distributed load (Han, 2007, 2009a,b). The force *dF* is generated by the pressure on small element *dζds* along its surface normal **n** (cos *n*, sin *n*):

(8) $$\begin{array}{l} dF=pd\zeta ds \end{array}$$

where,

(9) $$\begin{array}{l} d\zeta =dz-\left(\frac{\partial^{2}u_{c}}{\partial z^{2}}.r\cos\phi \right)dz=\left(1-\frac{\partial^{2}u_{c}}{\partial z^{2}}x\right)dz \end{array}$$

Its components in the *x* (buckling) direction and y (perpendicular to buckling) direction are:

(10) $$\begin{array}{r} dF_{x}=pd\zeta ds\cos n=pd\zeta dy\\ dF_{y}=pd\zeta ds\sin n=pd\zeta \left(-dx\right) \end{array}$$

By integrating along the circumference (inner wall) and dividing the central line length *dz*, the distributed load per unit vessel length generated by the lumen pressure is:

(11) $$\begin{array}{r} q_{x}=\oint p\frac{d\zeta }{dz}dy,\\ q_{y}=-\oint p\frac{d\zeta }{dz}dx \end{array}$$

Taking Equations (9) into (11) yields that:

(12) $$\begin{array}{r} q_{x}=p\oint \left(1-\frac{\partial^{2}u_{c}}{\partial z^{2}}x\right)\;dy=-pA_{lumen}\frac{\partial^{2}u_{c}}{\partial z^{2}};\;q_{y}=0 \end{array}$$

where *A*_*lumen*_ is the lumen cross-sectional area. Therefore, the laterally distributed load *q* generated by the internal pressure is in the *x* (buckling) direction. For circular cross-sections, $A_{lumen}=\pi \;r_{i}^{2}$ is the lumen radius), the equation becomes the same as previously reported (Han, 2007, 2009a,b).

#### Buckling Equation

With *q*_*x*_ (*z*) given in Equation (12), the buckling equation can be derived similarly as previously reported for vessels with a circular cross-section (Han, 2009a,b). Specifically, the general differential buckling equation becomes:

(13) $$\begin{array}{l} EI\frac{\partial^{4}u_{c}}{\partial z^{4}}+\left(pA_{lumen}-N\right)\frac{\partial^{2}u_{c}}{\partial z^{2}}=0 \end{array}$$

where *EI* represents the cross-sectional modulus and N is the axial tension (Han, 2009a,b). The critical pressure for pinned (*L*_*e*_ = *L*) or fixed ($L_{e}=\frac{L}{2}$) end support is:

(14) $$\begin{array}{l} p_{cr}=\frac{N+{\left(\frac{n\pi }{L_{e}}\right)}^{2}EI}{A_{lumen}} \end{array}$$

Where *n* represents buckling mode (Han, 2008, 2009a,b). This is the generalized lateral bent buckling equation for arteries with noncircular cross sections.

### Numerical Simulation of Noncircular Artery Buckling

Parametric studies of different levels of ovalness, eccentricity, and their combinations were performed using finite element simulations to illustrate their effects on the buckling behavior of arteries.

#### Model Geometries

Arteries were modeled as thick-walled cylindrical tubes with circular and elliptical lumens and outer walls. Various concentric and eccentric cross-sections were studied.

Concentric oval shape cross-sections with the major outer diameter, major lumen diameter, wall thickness, and vessel length of 6.99, 3.27, 1.86, and 52 mm (Lee et al., 2014), but of different minor diameters, were simulated and compared. A lumen aspect ratio (AR), defined as the ratio of the diameter in the direction of deflection (initial minor diameter) over the diameter in the direction perpendicular to the direction of deflection (initial major diameter), was used to describe the ovalness level of the lumen. Lumen AR in the range of 0.60–1.00 (with corresponding outer wall AR of 0.81–1.00) were analyzed. Since the opening angle has little effect on the critical pressure (Lee et al., 2012), we did not consider it in current simulations.

The eccentric oval-shaped cross-section models were built using the same lumen and outer wall dimensions as the concentric cross-section models. In this case, lumen AR of 0.80 and 1.00 with the eccentricity (*e* = ratio of the distance between the centers of the lumen and outer wall to the lumen major radius) equal to 20 and 40% of lumen major radius were considered. Lumen was moved alongside the direction of deflection, opposite, and vertical to the direction of deflection to create eccentricity in different directions to examine the directional effect.

#### Material Model

The arterial wall was assumed to be a homogeneous, anisotropic, incompressible material with Fung strain energy function (Chuong and Fung, 1986; Fung, 1993) in the form of:

(15) $$\begin{array}{l} w=\frac{C}{2}e^{Q} \end{array}$$

with

(16) $$\begin{array}{r} Q=b_{1}E_{\theta }^{2}+b_{2}E_{z}^{2}+b_{3}E_{r}^{2}+2b_{4}E_{\theta }E_{z}+2b_{5}E_{z}E_{r}\\ +2b_{6}E_{\theta }E_{r} \end{array}$$

where *E*_*r*_, *E*_θ_, and *E*_*z*_ are components of Green strain in the radial, circumferential, and axial direction, respectively, that are determined by the general equations for cylindrical coordinates (Fung, 1993; Humphrey, 2002). *b*_1_, *b*_2_, *b*_3_, *b*_4_, *b*_5_, *b*_6_, and *C*(kPa) are material constants obtained from experimental measurements and are equal to 0.679, 0.751, 0.228, 0.0519, 0.033, 0.051, and 20.42, respectively (Lee et al., 2014). In this study, we neglected the shear components in the Fung strain energy expression (Equation 16) based on the strain description given in Equation (6), which suggested that the shear components *E*_*rz*_ = *E*_θ_*z* = *0* and only *E*_*rθ*_ is nonzero. There is no data of b_7_ for *E*_*rθ*_ of porcine carotid artery available in the literature. Yu et al. (1993) reported that the shear modulus for *E*_*rθ*_ was very small for rat aorta segments subjected to bending. Therefore, we expect that the effect of the shear deformation would be small and negligible.

#### Finite Element Analysis of Bent Buckling

The lateral bent buckling behavior of arteries were simulated using the Static General Analysis in Commercial FEA package ABAQUS®. Arteries with elliptic cross-sections of different ARs described above were created using SolidWorks® and meshed using an 8-node linear hybrid hexahedral element with constant pressure (C3D8H). A mesh size of 0.2 mm was used after the pilot convergence study. A 1-degree initial curvature along the longitudinal axis was included in the models to facilitate buckling. Arteries were first stretched axially to the length of 67.6 mm (1.3 times of initial length) to mimic the *in vivo* axial stretch in the arteries (Lee et al., 2014). Then, a uniform static pressure (up to 50 kPa) was gradually applied to the vessel lumen. Both ends of the artery were restrained from rotations or axial move but allowed for radial displacement.

The eight intersection points of the minor and major diameter with the lumen and outer wall of the middle cross-section along the central axis of the vessel (at half of vessel length) where the deflection is maximum were selected as key points. The positions and displacements of these eight key points were monitored and used to determine the changes in AR, diameter, and deflection of the central axis. The onset of buckling is the time when the slope of the central axis deflection with respect to the pressure increases to 0.5 mm/kPa (at which the slope increase also speeds up).

## Results

### Deformation and Buckling of Arteries With Elliptic Cross Section

#### Change of Lumen Shape Under Axial Stretch and Lumen Pressure

**Figure 3:**
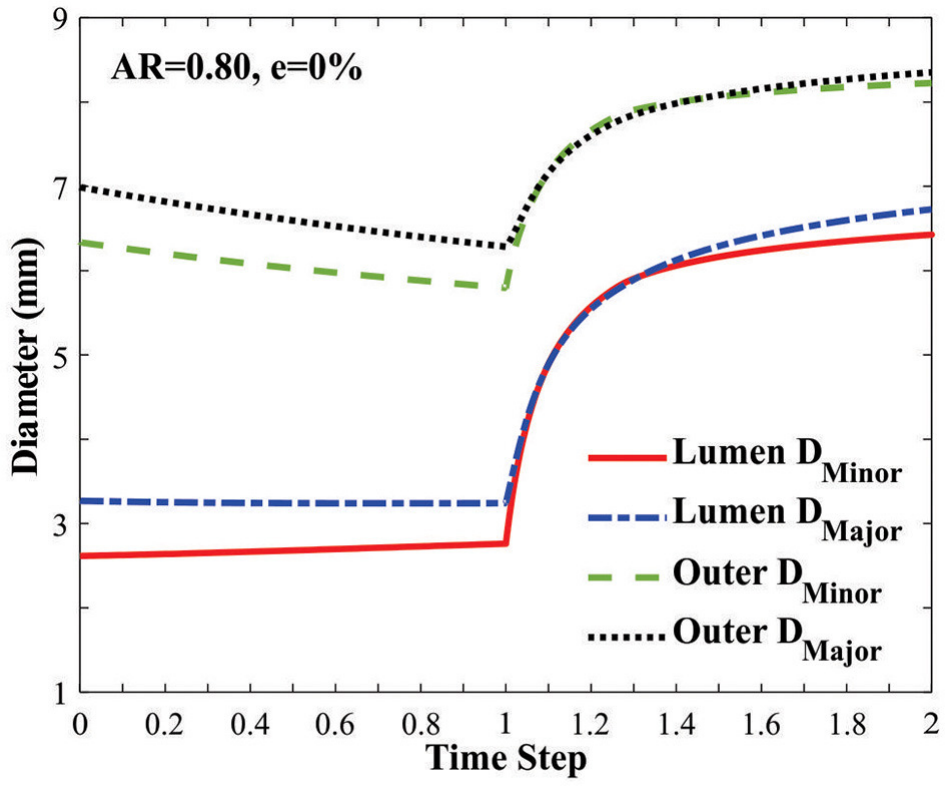
Changes of the minor and major diameters of the lumen and outer wall in an artery with an elliptic cross-section [initial AR = 0.80] under axial stretch (time steps 0–1.0 representing axial stretch ratio 1.0 to 1.3) and lumen pressure (time steps 1.0–2.0 representing lumen pressure 0–50 kPa). The vessel buckled at a lumen pressure of 12.74 kPa (at time step 1.25).

**Figure 4:**
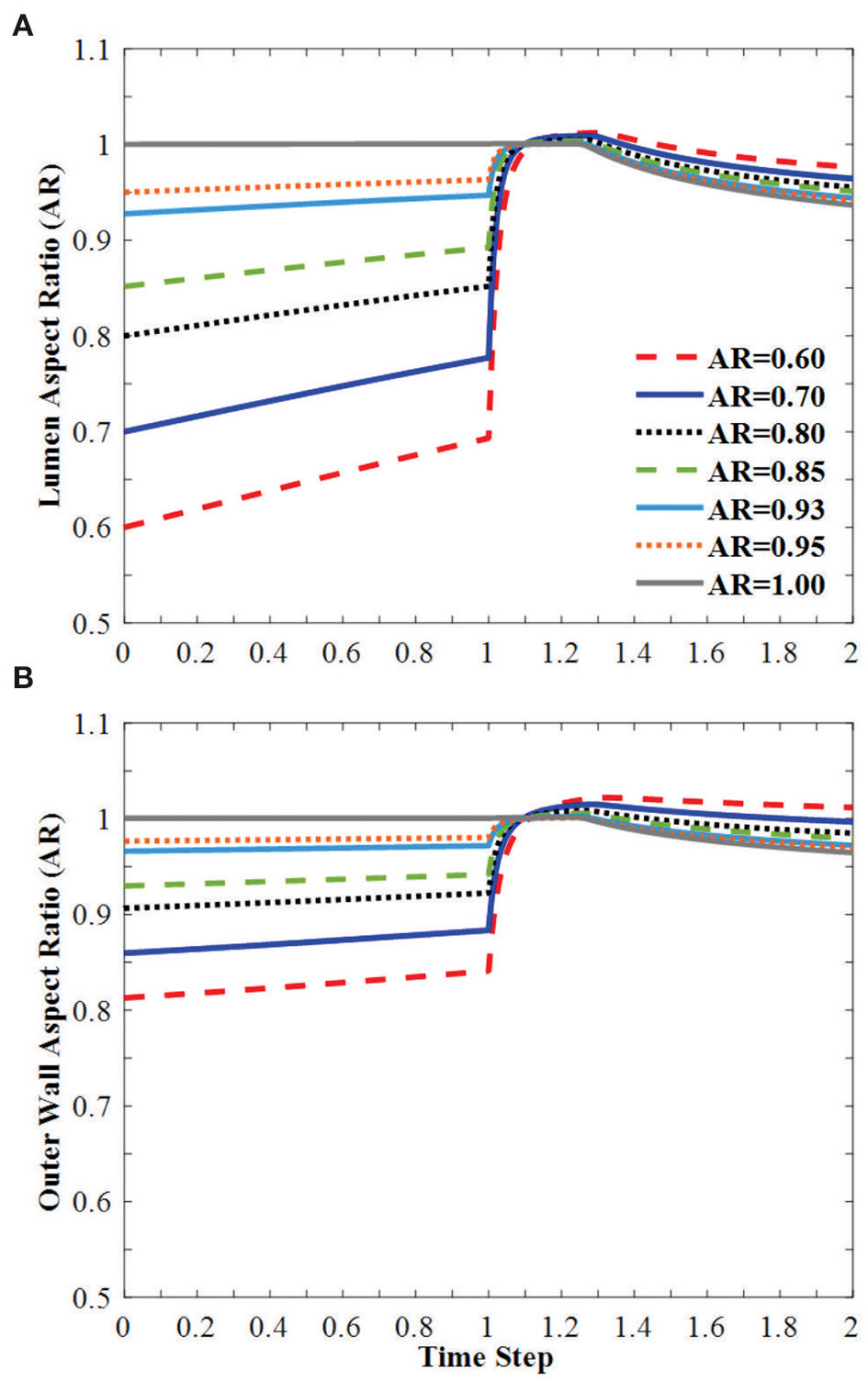
Change of **(A)** lumen AR and **(B)** outer wall AR of the middle cross-section of the vessel under axial stretch (time steps 0–1.0 representing axial stretch ratio 1.0 to 1.3) and lumen pressure (time steps 1.0–2.0 representing lumen pressure 0–50 kPa). The vessel buckles in the direction of the minor diameter. Buckling occurred at time step 1.25.

The vessel diameters changed under axial stretch and lumen pressure (Figure 3). It is seen that the minor lumen diameter increased with increasing axial stretch while the major diameter was nearly unchanged. Both of them increased with increasing lumen pressure. The outer wall minor and major diameters decreased with axial stretch (indicating wall thinning) but increased with increasing lumen pressure, even post-buckling. The corresponding changes in the aspect ratios of the lumen and outer wall of the middle cross section of arteries were observed under the applied axial stretch and pressurized inflation (Figure 4). The cross-sectional shape of the elliptic arteries tends to deform toward a circular shape under increasing lumen pressure, and then the lumen of the vessel becomes elliptic and collapses after buckling.

**Figure 5:**
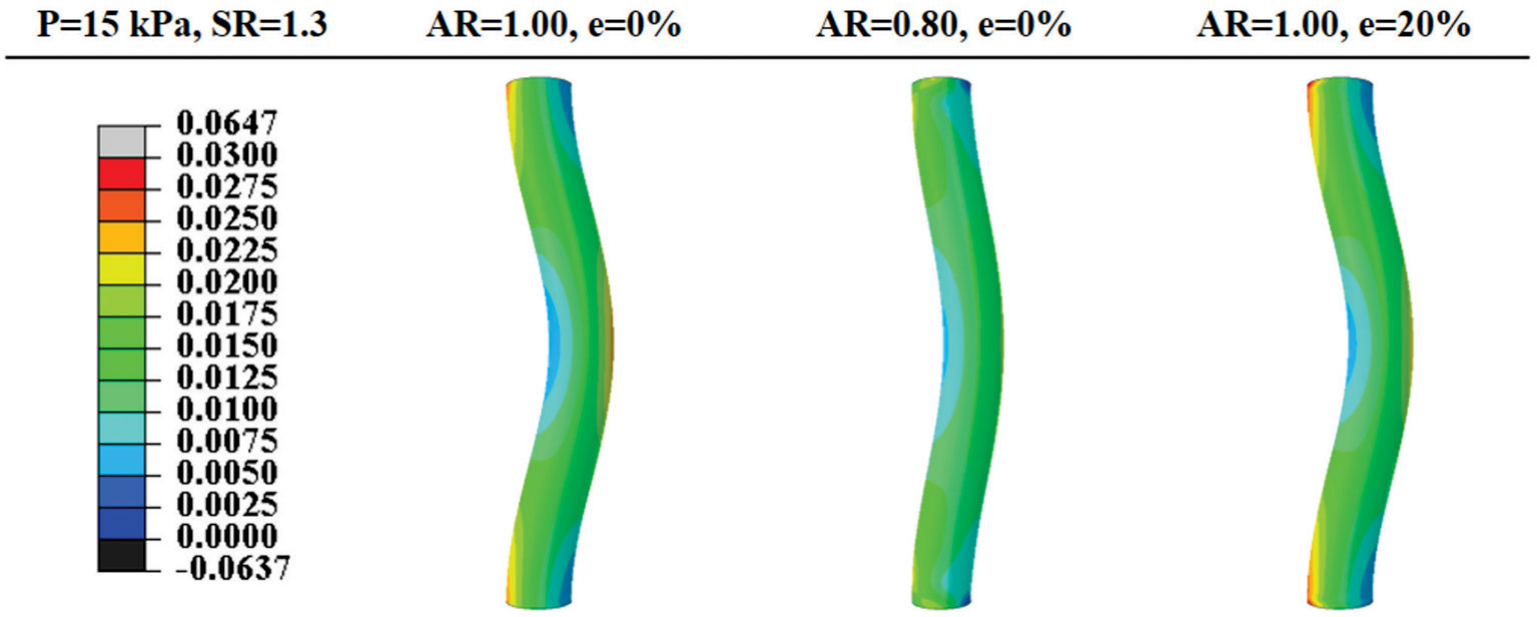
Comparison of post-buckling axial stress distributions in arteries with concentric circular and oval lumen shape and eccentric circular lumen shape (all under an axial stretch ratio of 1.3 and a lumen pressure of 15 kPa). Values of the color bar are in MPa.

Buckling mainly affects the axial stress distribution and has little effect on the radial and circumferential stress distributions. The axial stress was elevated in the convex side of the vessel and reduced on the concave side (Figure 5).

The initial curvature was applied in different directions (along the minor or major diameters) to trigger buckling in that direction for oval-shaped arteries. The critical pressure for buckling in the direction of minor diameter was less than the critical pressure for buckling in the direction of major diameter. Therefore, buckling for the oval arteries will occur in the direction of its minor axis of the lumen.

#### The Relation Between AR and the Critical Pressure

**Figure 6:**
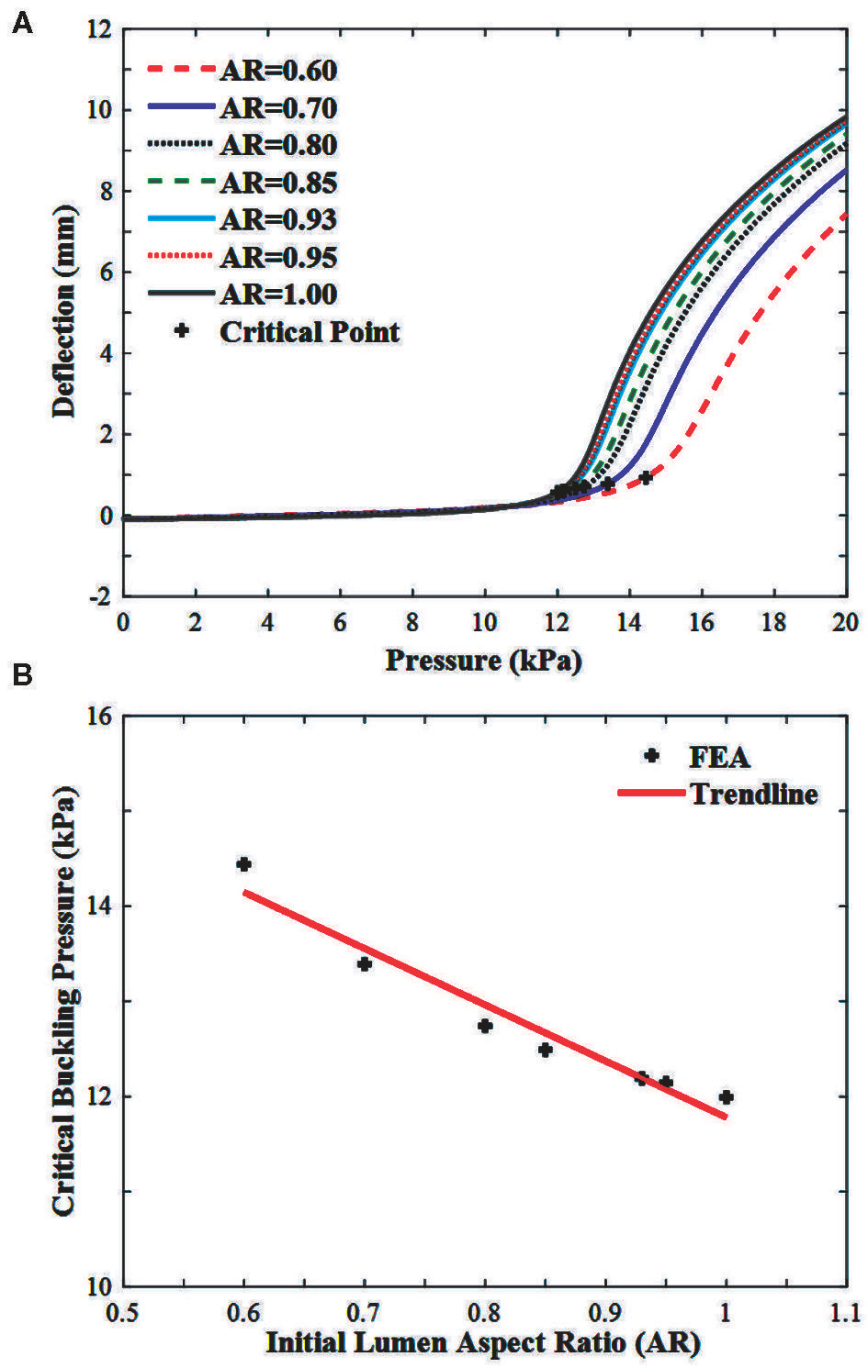
Buckling of arteries with concentric elliptical cross sections. **(A)** Deflections plotted as functions of lumen pressure. The critical buckling points are marked by “+”. **(B)** Critical buckling pressure plotted as a function of lumen aspect ratio in the range of 0.60–1.00.

All arteries buckled when the lumen pressure reached a critical pressure. For arteries with an initial lumen AR in the range of 0.60–1, the critical pressure increases linearly with decreasing AR (Figure 6). The critical pressure increased by 20.4% when the initial lumen AR changes from 1 to 0.60.

### Deformation and Buckling of Arteries With Eccentric Cross Section

#### Change of Lumen and Outer Wall Shape Under Axial Stretch and Lumen Pressure

**Figure 7:**
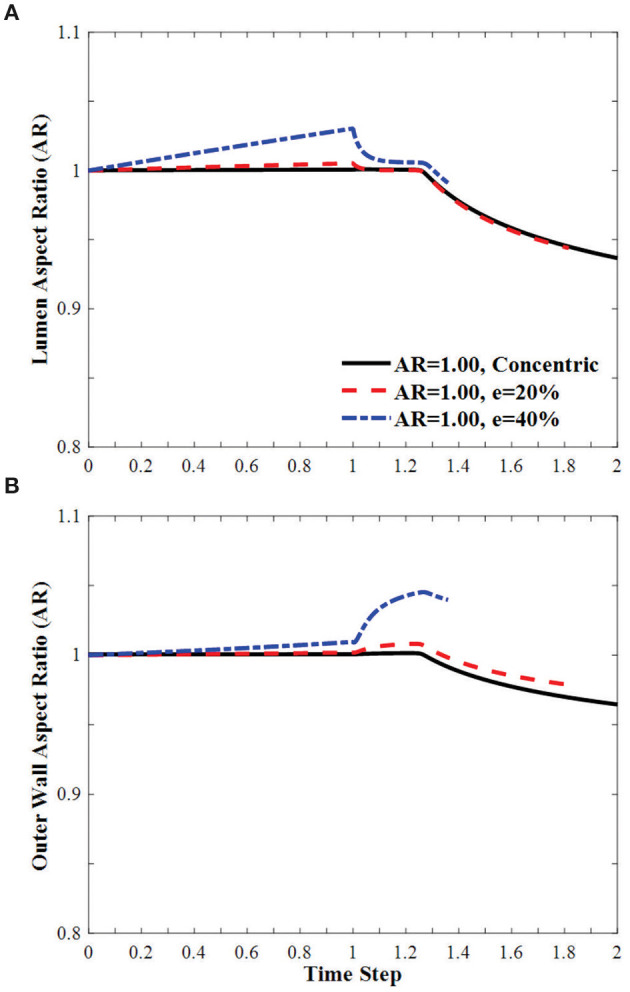
Effect of eccentricity on the AR of the **(A)** lumen and **(B)** the outer wall of circular arteries (initial AR = 1) during the axial stretch and lumen pressure loading process. Initial eccentricity (e) of 0% (concentric), 20 and 40% in the direction opposite to the direction of deflection is compared. The AR is calculated as the ratio of diameter in the buckling direction vs. the diameter in the direction vertical to the buckling direction. Time step is the same as in Figure 3.

In arteries with circular lumen and outer wall at a given initial eccentricity of 20 and 40%, the ARs of the lumen becomes oval shape under axial stretch, changed toward circular shapes with increasing lumen pressure and become oval shape post-buckling (Figure 7). The AR of the arteries with eccentricity becomes higher than the artery with concentric cross-section throughout the loading process (axial stretching and pressurization). The AR of the outer wall changes more dramatically with lumen pressure and the effect is elevated as the initial eccentricity of the artery increases.

#### Effect of Eccentricity on the Critical Pressure

**Figure 8:**
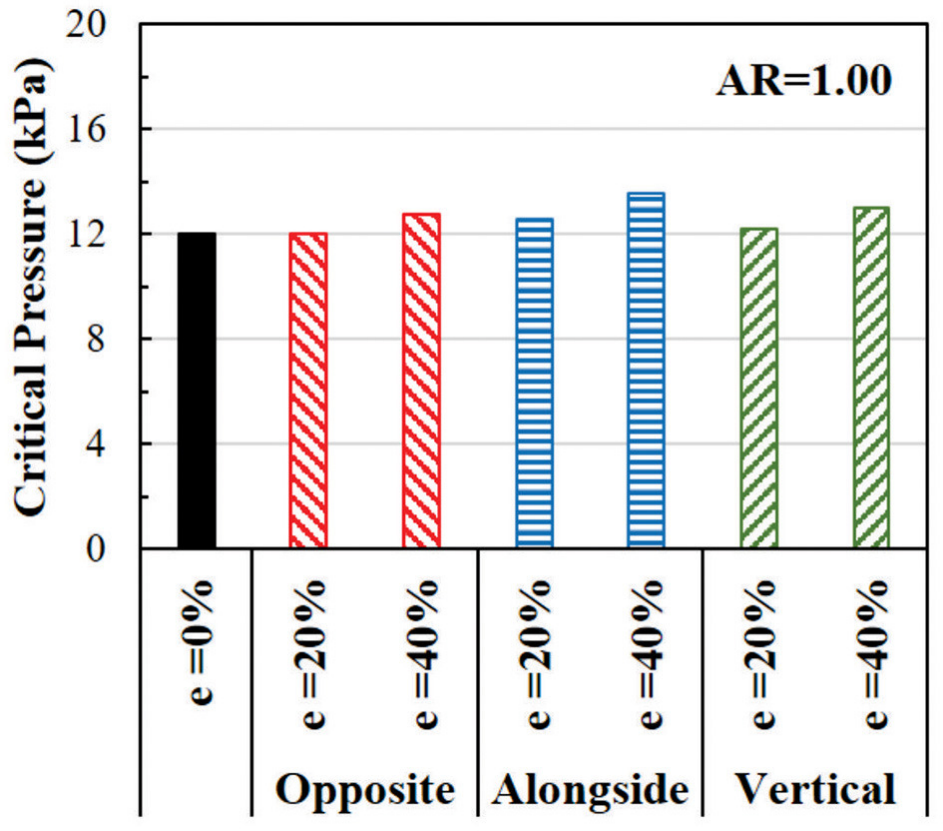
Comparison of the critical buckling pressures of arteries with eccentric cross-section. The effect of lumen eccentric alongside (same), opposite, and vertical to the direction of deflection are compared.

The effect of eccentricity on the critical pressure of arteries is illustrated in Figure 8. The critical pressure increases when the lumen is eccentric. The higher level of eccentricity (40%) has a larger effect than the low eccentricity (20%). The effect of eccentricity varies when the eccentric direction (from the center of the outer wall to the center of the lumen) changes relative to the buckling direction. The largest effect is seen when the eccentric direction is alongside the buckling direction, while the smallest effect is when the eccentric direction is opposite to the buckling direction. Therefore, eccentric arteries buckle in the direction opposite to its eccentric direction.

### Combined Effects of Ovalness and Eccentricity

**Figure 9:**
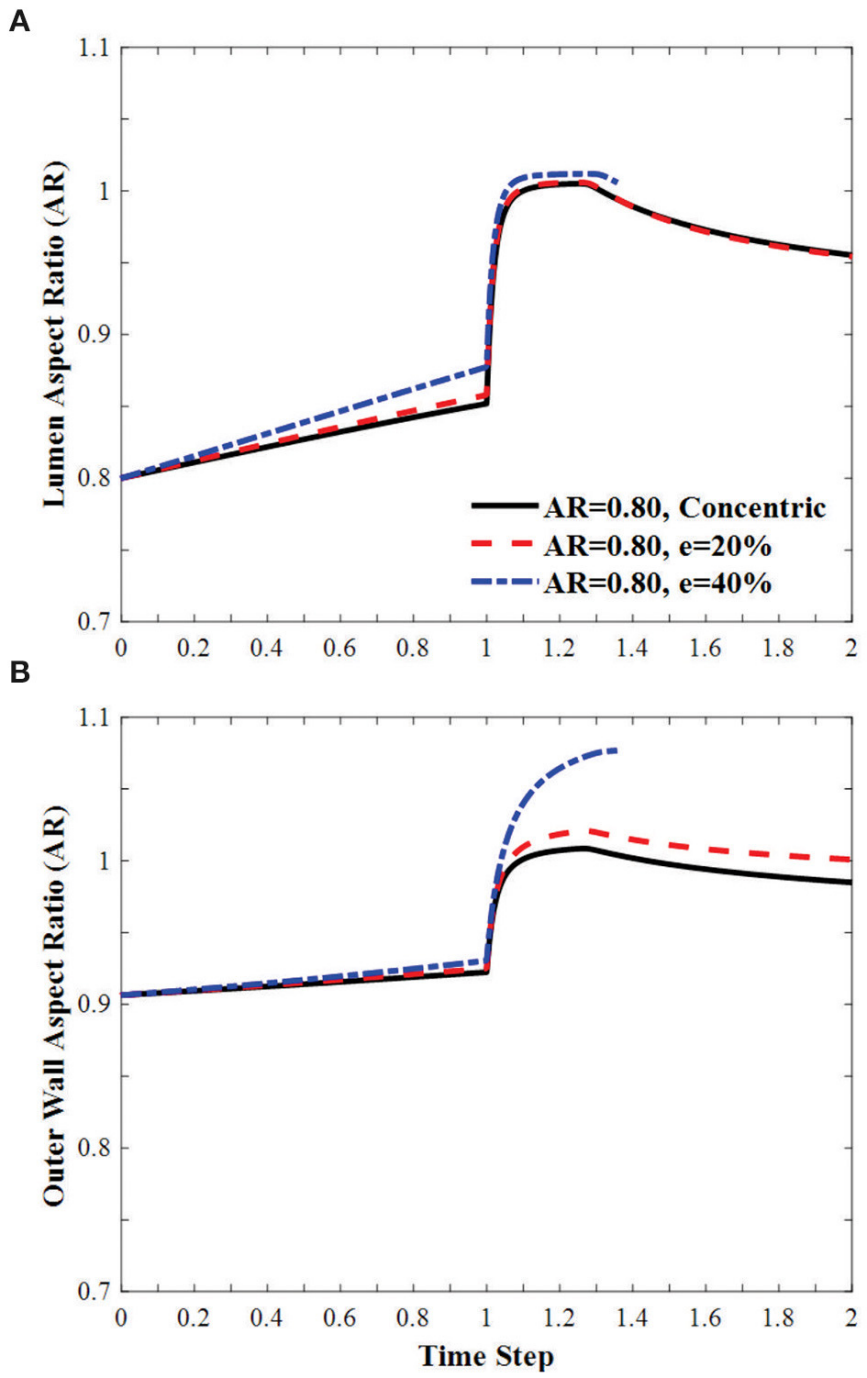
Effect of eccentricity on the AR of the **(A)** lumen and **(B)** the outer wall of elliptic arteries with initial lumen AR = 0.8 during the axial stretch and lumen pressure loading process. Initial eccentricity (e) of 0% (concentric), 20 and 40% in the direction opposite to the direction of deflection. The AR is calculated as the ratio of diameter in the buckling direction vs. the diameter in the direction vertical to the buckling direction. Time step is the same as in Figure 3.

**Figure 10:**
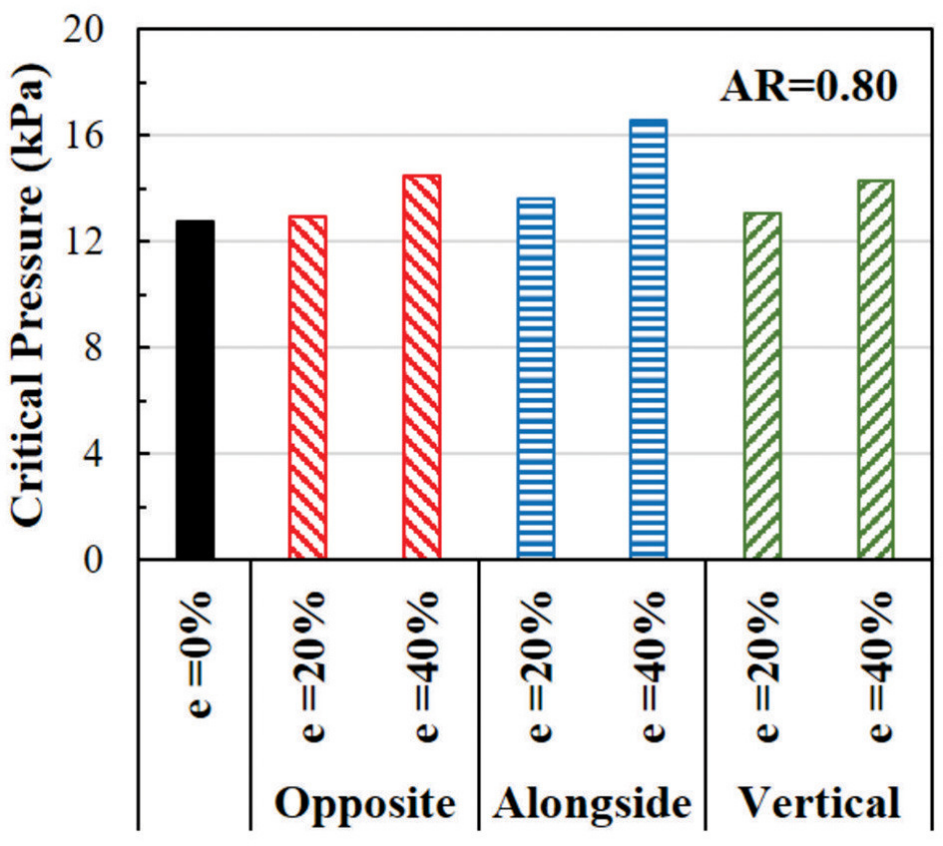
Comparison of the critical buckling pressures of arteries with an eccentric elliptical cross-section with initial lumen AR = 0.80. The critical pressure of arteries with lumen eccentric alongside (same), opposite, and vertical to the direction of deflection are also compared.

In oval arteries at a given initial AR = 0.80 and eccentricity of 20 and 40% opposite to the direction of deflection, the ARs of the lumen of the middle cross-section of eccentric arteries changed toward circular shapes with increasing lumen pressure and become oval post-buckling (Figure 9). The AR of these arteries becomes higher than the artery with concentric cross-section throughout the loading process (axial stretching and pressurization). When the lumen is eccentric alongside, opposite, or vertical (sideways) to the direction of deflection, the critical pressure becomes slightly higher than the concentric arteries (Figure 10). The vessel eccentric to the direction opposite to the direction of deflection has the lowest critical pressure among the three. These results indicate that the artery will buckle in the direction opposite to the direction of eccentricity. In addition, increasing the level of eccentricity enhances these effects. It is seen that decreasing AR and increasing eccentricity simultaneously affect the critical pressure of the artery more than each factor alone.

## Discussion

In this study, we developed the theoretical buckling equation for arteries with noncircular cross-sections and illustrated the effects of oval and eccentric cross-sections on the critical buckling pressure of arteries. The results demonstrated that arteries with concentric elliptical cross-section buckle in the direction of the minor diameter. The elliptic lumen and outer wall deform toward circular shapes with increasing lumen pressure and then deform back to be more elliptic post-buckling. The critical pressure of the elliptic vessel increases with the reduction of the initial lumen AR. Similarly, eccentricity also increases the critical pressure of arteries, and more increase is seen in vessels with a higher level of eccentricity and even more, the increase is seen in vessels with combined ovalness and eccentricity. The theoretical equations proved that artery buckling theory could be expanded to noncircular vessels and the numerical simulations illustrated the effects of ovalness and eccentricity. These two approaches are complementary to each other.

The ranges of the ovalness and eccentricity used in the simulation cover the range we have seen in mammalian arteries. Estimations using previously reported data (Han and Fung, 1991, 1996) showed that the lumen AR and eccentricity of porcine aorta varied 0.5–1.0 and 0–14%, respectively. Similarly, oval cross-section and eccentricity have been seen in the human aorta, coronary, and carotid arteries. For example, AR in the range of 0.55–1 and 0.65–1 have been reported for human coronary and carotid arteries, respectively (Zeina et al., 2007; Kamenskiy et al., 2012). An elliptic cross-section is often seen in the aneurysmal abdominal aorta as well (Kyriakou et al., 2020), and atherosclerotic plaque often leads to local eccentricity in stenotic arteries in which the eccentricity can reach up to 200% of the lumen radius (Aoki and Ku, 1993; Ohara et al., 2008).

The critical pressure of the artery with circular cross-section obtained from FEA in this study matched well with previous experimental results (Lee et al., 2012) and theoretical model simulation (12 kPa vs. 10 kPa and 10.5 kPa, respectively). It provides validation for the simulation methods. Due to the use of initial curvature as imperfection to trigger buckling, arteries may gradually reach a deflection of 0.5 mm under lumen pressure before buckling. Therefore, we used rate of deflection increase which reflects the loss of stability to determine the critical pressure in this study. This is different from previous studies (that used 0.5 mm deflection) and could be a possible reason for the difference in the FEA results of oval and eccentric arteries (Datir et al., 2011).

Circular arteries may buckle laterally toward arbitrary directions. Oval arteries, however, due to the difference in the bending rigidity EI, tends to buckle in the short axis (minor diameter) direction. This is because that EI is lower in the minor diameter direction. While the cross-section is very close to circular under lumen pressure, the stress could be slightly higher when bending along the major diameter. Due to material nonlinearity, the corresponding EI could be higher and thus the critical buckling pressure in the direction. Similarly, for both oval and eccentric vessels, the increase in EI could be the reason for increased critical buckling pressure.

### Limitations

There are a few limitations to this study. First, the arterial wall was assumed to be composed of homogenous material and the variation along the three layers of the arterial wall was ignored (Mottahedi and Han, 2016). Second, the arterial wall was assumed to be uniform along its axial length and possible variations such as tapering were ignored (Datir et al., 2011; Lee et al., 2014). Third, static pressure was used and the possible dynamic buckling effects were not considered (Rachev, 2009; Liu and Han, 2012). These factors need to be considered in future studies. Despite these limitations, the current study increases the understanding of the stability of arteries with a concentric and eccentric elliptical cross-section.

Besides bent buckling, twist buckling and helical buckling may occur in arteries and veins (Selvaggi et al., 2006; Wong et al., 2007; Garcia et al., 2013, 2017; Rodriguez and Merodio, 2016; Sharzehee et al., 2019). Barrel shape bulging buckling and cross-sectional collapse and “beads” -like or “aneurysmal” buckling may also occur as shown in recent reports (Alhayani et al., 2013; Emery and Fu, 2021; Font et al., 2021; Fu et al., 2021). It could be interesting to explore how ovalness and eccentricity affect artery twist, collapse, and helical buckling in future studies.

### Significance and Clinical Relevance

Arteries *in vivo* may become twisted or tortuous (Han, 2012; Garcia et al., 2013). It has been shown recently that artery buckling may occur due to reduced axial tension, excessive blood pressure, or elastin degradation (Jackson et al., 2005; Lee et al., 2012; Zhang et al., 2014). Buckled arteries could become tortuous or kinked, which disturb or disrupt the normal blood flow and alter the wall stress distribution and lead to wall remodeling (Xiao et al., 2014; Zhang et al., 2014; Wang et al., 2015; Weiss et al., 2020). The current results demonstrated that geometric variations such as oval and eccentric cross-section, which occurs in many blood vessels, may increase the critical pressure and, hence, make arteries more stable than circular cylindrical arteries.

Understanding the underlying biomechanics of artery tortuosity can be useful in vascular physiology, pathology, and surgery. The current results broaden the understanding of vascular biomechanics and shed light on the stability and tortuosity of blood vessels.

## Data Availability Statement

The raw data supporting the conclusions of this article will be made available by the authors, without undue reservation.

## Author Contributions

YS: conducted the simulations, analyzed the data, and wrote and reviewed the manuscript. H-CH: designed the study, derived the theoretical equations, analyzed the data, and wrote and reviewed the manuscript. All authors contributed to the article and approved the submitted version.

## Conflict of Interest

The authors declare that the research was conducted in the absence of any commercial or financial relationships that could be construed as a potential conflict of interest.

## Publisher's Note

All claims expressed in this article are solely those of the authors and do not necessarily represent those of their affiliated organizations, or those of the publisher, the editors and the reviewers. Any product that may be evaluated in this article, or claim that may be made by its manufacturer, is not guaranteed or endorsed by the publisher.
